# Development of a Vision-Guided Shared-Control System for Assistive Robotic Manipulators

**DOI:** 10.3390/s22124351

**Published:** 2022-06-08

**Authors:** Dan Ding, Breelyn Styler, Cheng-Shiu Chung, Alexander Houriet

**Affiliations:** 1 Human Engineering Research Laboratories, VA Pittsburgh Healthcare System, Pittsburgh, PA 15206, USA; brs251@pitt.edu (B.S.); joshua.chung.cs@pitt.edu (C.-S.C.); ach75@pitt.edu (A.H.); 2 Department of Rehabilitation Science and Technology, University of Pittsburgh, Pittsburgh, PA 15213, USA; 3 Department of Bioengineering, University of Pittsburgh, Pittsburgh, PA 15213, USA

**Keywords:** upper-limb impairments, activities of daily living, multi-step tasks, semi-autonomous control

## Abstract

Assistive robotic manipulators (ARMs) provide a potential solution to mitigating the difficulties and lost independence associated with manipulation deficits in individuals with upper-limb impairments. However, achieving efficient control of an ARM can be a challenge due to the multiple degrees of freedom (DoFs) of an ARM that need to be controlled. This study describes the development of a vision-guided shared-control (VGS) system and how it is applied to a multi-step drinking task. The VGS control allows the user to control the gross motion of the ARM via teleoperation and commands the ARM to autonomously perform fine manipulation. A bench-top test of the autonomous actions showed that success rates for different subtasks ranged from 80% to 100%. An evaluation with three test pilots showed that the overall task performance, in terms of success rate, task completion time, and joystick mode-switch frequency, was better with VGS than with teleoperation. Similar trends were observed with a case participant with a spinal cord injury. While his performance was better and he perceived a smaller workload with VGS, his perceived usability for VGS and teleoperation was similar. More work is needed to further improve and test VGS on participants with disabilities.

## 1. Introduction

People with upper-limb impairments due to neuromuscular conditions (e.g., high-level spinal cord injury and amyotrophic lateral sclerosis) or other physically disabling conditions often have difficulty performing activities of daily living (ADLs) that require object-handling and -manipulation. Assistive robotic manipulators (ARMs) have emerged as a potential solution to mitigate the difficulties, frustration, and lost independence experienced by these individuals [1,2]. ARMs can be mounted on a mobile platform or a wheelchair, potentially providing daily assistance over a variety of ADLs (e.g., eating, drinking, personal care, household chores, and school/work-related activities) and accommodating people with a wide range of diagnoses [1,2]. However, achieving effective and efficient control of an ARM can be a challenge. One problem is that an ARM is often equipped with 6–7 degrees of freedom (DoFs), but conventional joysticks or switch controls have 1–2 DoFs. For example, to fully control the end-effector of an ARM in three linear positions (x, y, and z) and three angular positions (yaw, pitch, and roll), the user has to constantly switch between four control modes when using a conventional 2-DoF joystick along with a switch button(s). Such operation becomes unintuitive and tedious, especially when the ARM gets close to the target and needs constant adjustment to align appropriately for manipulation [3,4].

Research in this area has focused on developing new ways to control ARMs, including new control interfaces [5,6], remapping control inputs [7], and shared-control schemes [3,8]. Most of the work on new control interfaces has aimed to provide an alternative means of ARM control such as voice control [9], gaze control [10,11], tongue control [12], brain–computer interface control [13], body movement control via inertia measurement units (IMUs) [14], and gesture control via computer vision [15]. These new control interfaces were often reported as a proof-of-concept, with limited or no information on their effectiveness and efficiency for different manipulation tasks. In terms of remapping control inputs, Losey et al. discovered that the traditional control inputs for the 6-DoF linear and angular velocity of the end-effector could be captured by 2-DoF latent actions for the pouring of water into a glass task, i.e., the action of carrying the cup level with the table and performing a pouring action [7]. The study showed that this remapping approach led to a greater success rate, faster completion time, and less effort than teleoperation or shared control. However, low-DoF latent actions need to be learned from task-specific training data, and participants did not indicate a preference for this approach. For example, the user might desire more freedom of control. Additionally, it is also unclear how intuitive the control is for robot configurations that are not addressed during the training phase.

Shared control in ARMs has been implemented in two ways: (1) blending autonomy—control from the user and control from the ARM are blended to complete a task; and (2) task allocation—the user and the ARM are responsible for different parts of a task.

- In terms of blending autonomy, research has focused on human intent recognition [16] as well as different strategies to blend user and robot control for shared autonomy. For example, early work on the MANUS manipulator discussed the scheme of allowing the robot and user to control different DOFs (e.g., allowing the user to control the end-effector linear position and the robot to control the end-effector pose) [17]. Gopinath et al. proposed a blending scheme for the velocity of the robot end-effector in Cartesian space that can be tuned for the level of robot assistance [18], and found that the custom assistance was not always optimized for task performance, because some participants favored retaining more control over better performance. In general, research on blending autonomy has mostly focused on intent recognition accuracy and optimal blending schemes instead of its practical applications towards complex multi-step tasks.
- In terms of task allocation, the user and robot are each assigned a certain part of a task to perform. For example, Bhattacharjee et al. implemented a fully functional robot-assisted feeding system by allocating high-level decision-making tasks to the user (e.g., which food item to pick, how the food item should be picked up by the robot, when and how the robot should feed the user) via a touchscreen and allocating all motion planning and control to the robot, without requiring the user to teleoperate it. While user performance (which was not a focus of the study) was not reported, the system was well-received by the participants with disabilities, with relatively high perceived-usefulness and ease-of-use ratings. Our group developed a shared-control system whereby we designated the user to control the gross motion of the arm via teleoperation and the robot to take over the fine manipulation autonomously when getting close to the target object. We evaluated the system with eight individuals with disabilities and found that it improved task completion time and reduced perceived workloads for all five tasks tested. However, the five tasks, including turning a door handle, flipping a light switch on/off, turning a knob, grasping a ball, and grasping a bottle, were discrete tasks that require one-step operation [19].

In this paper, we extend our previous shared-control approach [16] to address multi-step functional tasks. We describe the system design, its implementation over a multi-step drinking task, and the preliminary evaluation results.

## 2. Materials and Methods

### 2.1. The Robot

**Figure 1:**
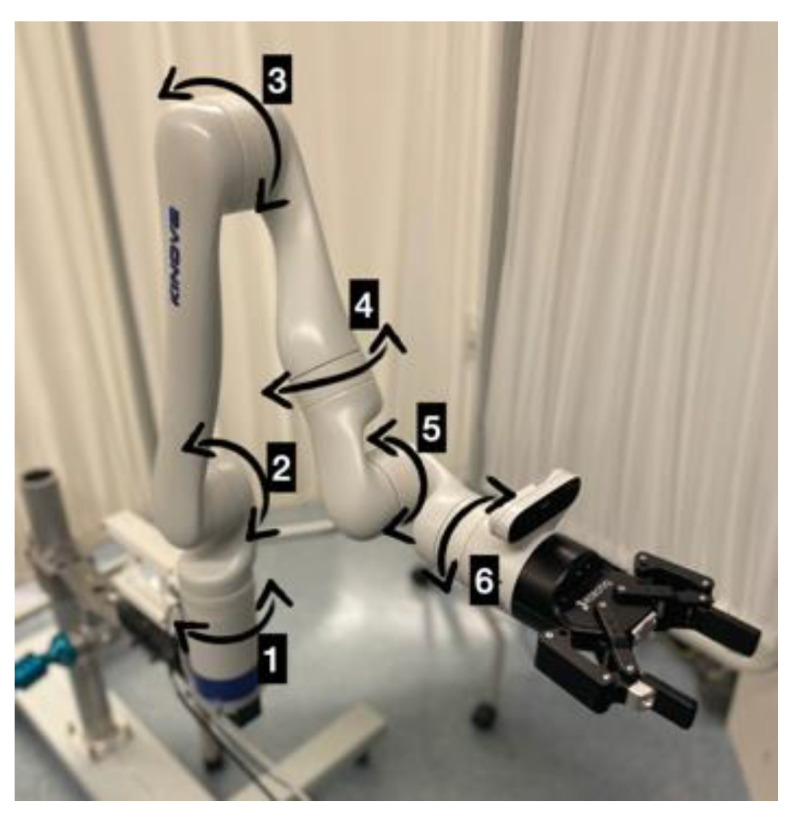
The robot with joints labelled (1–6) and arrows showing rotation for each of the six joints.

Our setup consists of a 6-DoF Kinova Gen 3 robotic arm (Kinova Inc., Boisbriand, QC, Canada) with a 2-finger Robotiq gripper of 85 mm stroke (Robotiq, Levis, QC, Canada) (Figure 1). The robotic arm (without the gripper) weighs 7.2 kg (or 16 lbs) and has a continuous payload of 4 kg (or 8.8 lbs) and a maximum reach of 891 mm (or 35 inches). It is also equipped with a vison system at the wrist which includes a color sensor and a depth sensor (Intel^®^ RealSense^TM^, Santa Clara, CA, USA).

### 2.2. Vision-Guided Shared-Control (VGS) System

**Figure 2:**
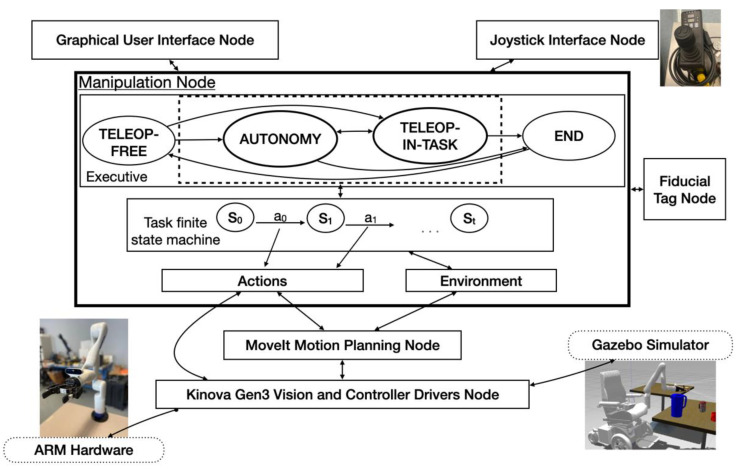
VGS software system architecture.

The VGS control includes three components, i.e., user teleoperation, autonomous robot operation, and control-authority transition between the user and the robot. The system always put the user in charge, allowing him/her to control the gross motion of the robot via teleoperation, and to command the robot to autonomously perform more challenging fine manipulation, which often requires not only a good view of the gripper interacting with the target object, but also precise alignment. The software architecture of the VGS control is shown in Figure 2. The software system runs Ubuntu 18.04 on a NVDIA^®^ Jetson AGX Xavier computer and uses the Robot Operating System (ROS) Melodic Morenia publish–subscribe architecture to asynchronously send information between processes referred to as nodes.

**Figure 3:**
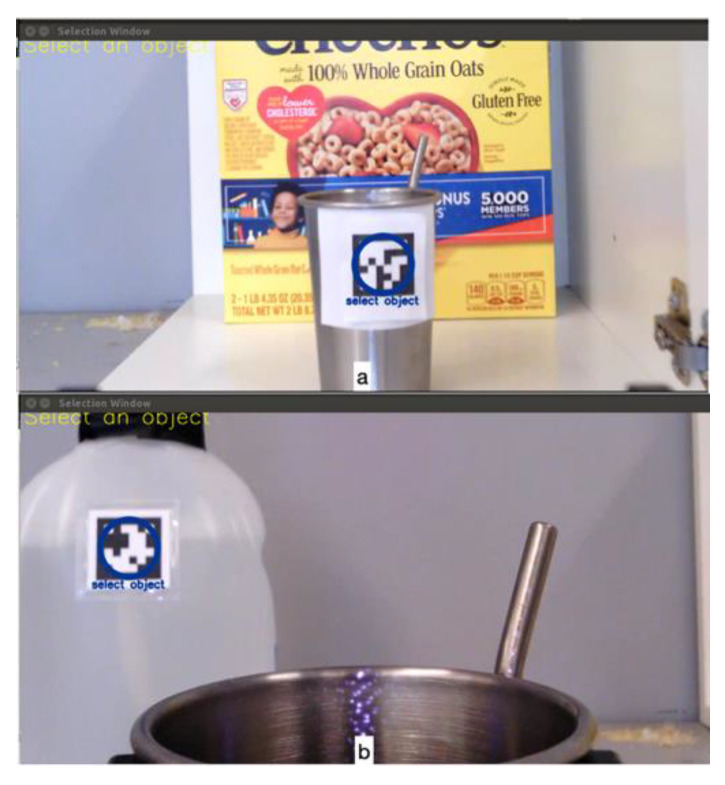
The touchscreen displays an object selection GUI overlayed on the gripper’s camera view. (**a**) shows tag detection with a blue selection circle highlighted to initiate a cup grasp, and (**b**) shows a selectable blue circle that initiates automatically moving the ARM to a jug fill position.

**Figure 4:**
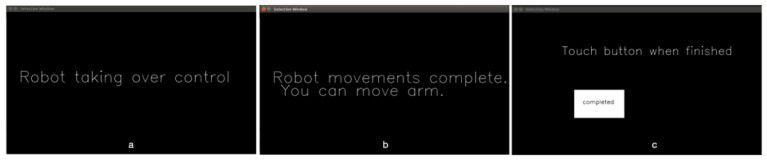
Messages displayed on GUI. (**a**) is the message displayed on the touchscreen during the *Autonomy* state. (**b**) is displayed once autonomous control has finished and transitions to the user controlled *Teleop Free* state. (**c**) is the message displayed during *Teleop in Task* where the system waits until the user is done with a temporal operation (i.e., filling the cup with water, or drinking from the cup) before transitioning back to *Autonomy*.

- User teleoperation is achieved through the joystick interface node. This node supports different types of joysticks including an X-box game controller, a 3-DoF joystick, and a traditional 2-DoF joystick. For example, a 2-DoF joystick along with two buttons (either on the joystick or external switches) is configured with one button for switching between Mode 1 (end-effector translational movements in forward/backward and left/right directions) and Mode 2 (end-effector translation movements in up/down directions and wrist roll rotations), and another button for switching between Mode 3 (wrist orientation in pitch and yaw directions) and Mode 4 (gripper open/close). For a 3-DoF joystick, it is also configured with two buttons, with one button for switching between Mode 1 (end-effector translation) and Mode 2 (end-effector orientation), and the other button for switching to Mode 3 (gripper open/close).
- Autonomous robot operation is initiated through the fiducial tag node. Fiducial tags offer highly distinguishable patterns with strong visual characteristics, and are often used for the identification, detection, and localization of different objects. We chose ArUco tags in this study given their great detection rate, good position and orientation estimation, and low computational cost [20]. The fiducial tag node wraps an open source ArUco library [21,22] into the ROS architecture for publishing the number, position, and orientation of each ArUco tag fixed to a target object with respect to the robot’s wrist-mounted camera. The information is published to the manipulation node and used to display a tag selection area on the graphical user interface (GUI), and to model obstacles in the environment.
- The manipulation node contains the main system state-machine, referred to as the system executor. When the VGS control is started, the system executor runs continuously for the system’s lifetime. This node contains a configuration file written in the YAML data-serialization markup language, for defining object properties including shape, size, and position relative to the ArUco tag. The node also supports the system state transitions between autonomous and user-teleoperation actions. The system executor calls various actions for each subtask that are pre-defined in an action library. For autonomous actions, once the user selects an object for interaction, the manipulation node parses the shape and size of the environment obstacles based on the ArUco tag ID. Obstacles are then added to the environment-planning scene for the robot path planning. The VGS automatically moves the robotic arm, through software, to a 6-DoF goal pose in the environment that is either achieved through motion planning or through a direct call to the Kinova Kortex driver. In both software calls, the third-party libraries (MoveIt ROS package or Kortex ROS driver package) both perform an inverse kinematic calculation utilizing their own internal kinematic solvers. A MoveIt motion-planning framework [23,24] is used to generate obstacle-free paths. The path is first generated by solving for an inverse kinematic solution using the Trac IK kinematic plugin. If a solution is not found, the planning then tries to generate a feasible path using the sample-based RRTConnect planner [25]. If all planners fail to find a solution, the robot goes back to the home position, re-plans or aborts the autonomous action, and prompts the user to teleoperate the robot. Successful paths are then executed through calls to the Kinova Gen 3 controller, which moves the arm to specific path positions. Actions that do not require motion planning, such as opening the gripper and pulling to open the cabinet, directly call the Kortex driver. As different parts of the task share common actions (e.g., reaching for an object), the executor chooses an action to call (sometimes repeated) based on a state machine that describes the current subtask.
- The control-authority transition between the user and the robot is achieved through the GUI node. A touchscreen is placed in front of the user for target selection. This screen also keeps the user informed during the control-authority transition via text messages displayed on the screen. The transition happens between three system states. The *Autonomy* state is when the robot has full control of the system and automatically moves the arm. The *Teleop Free* state is when the user has joystick control, and the system is not in a task action. Lastly, the *Teleop in Task* state is when the user has joystick control, and the system is within a task action. Depending on the current system state, the GUI screen changes. For example, Figure 3 shows two examples during *Teleop Free* where a selectable circle appears over the fiducial tag for interacting with the cup (Figure 3a) or interacting with the water jug (Figure 3b). Figure 4 shows messages displayed to the user during *Autonomy* (Figure 4a), at the completion of *Autonomy* (Figure 4b), and during *Teleop in Task* (Figure 4c).

### 2.3. Experiment

**Figure 5:**
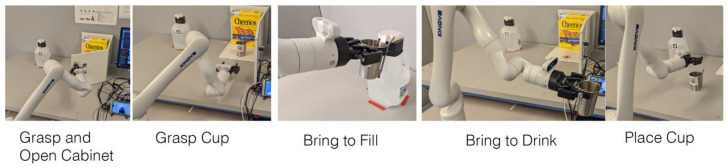
Subtasks for a drinking task.

We implemented the VGS control for a multi-step drinking task, which consisted of five subtasks: opening a cabinet, retrieving a cup, filling the cup with water, drinking from the cup, and placing the cup back on the table (Figure 5).

As shown in Figure 5, the subtasks required different manipulation types such as “pull” for opening a cabinet, “pick” for retrieving a cup, “push” for filling the cup with water, and “place” for putting the cup back on the table. For opening a cabinet, we added a 1” tube over the original ½” cabinet door handle to facilitate grasping, with a larger target for both teleoperation and VGS control. This was also helpful for accommodating positioning errors associated with fiducial tag detection for the VGS control.

The whole VGS control process is described as follows: For opening a cabinet, the user teleoperates the robot until it sees the fiducial tag on the cabinet. Once the user selects the tag on the touchscreen, the robot will take over the task of grasping the cabinet handle and opening the cabinet fully. The system then prompts the user to take control for the next step—retrieving a cup—through a message on the touchscreen. The user teleoperates the robot to find the tag on the cup, and then selects the tag on the touchscreen to command the robot to autonomously grasp the cup and lift it up. The system then prompts the user to move on. The user teleoperates the robot with the cup in hand until it finds the tag on the jug and selects the tag. The robot then autonomously moves close to and aligns the cup with the dispensing tap of the jug, and the system prompts the user to fill the cup with water via teleoperation. This way, the user can control the amount of water he/she will need. The system then prompts the user to bring the cup to a default drinking position by tapping a button on the touchscreen. The user can further adjust the robot’s position via teleoperation until he/she can drink from the cup. The system prompts the user to place the cup back on the table by tapping a button on the touchscreen. Table 1 summarizes the user and robot actions for the drinking task under the VGS control and the criteria for task success.

We first performed a bench-top test of the autonomous part of the VGS control under each subtask. During the test, the Kinova Gen 3 robotic arm was mounted on a table placed in front of the experimental setup. For each autonomous action, we started the robot in an arbitrary position around the target object (e.g., left, right, and in front), and commanded it to complete the action by selecting the object on the touchscreen. For opening the cabinet, 20 trials were performed with a fixed initial gripper-orientation (parallel to the table and perpendicular to the cabinet door) and an additional 20 trials were performed at arbitrary initial gripper-orientations. The other subtasks were also tested for 20 trials each from a fixed initial gripper-orientation. The success rates and reasons for failure were recorded.

We then had three test pilots without disability, who are also part of the research team, trial the two control methods: teleoperation and VGS control. Two test pilots had very limited experience operating a robotic arm, and one had significant experience. The experiment setup was similar to the bench-top test. The robot was mounted on a table placed in front of the experimental setup, and the test pilot sat to the right of the robot. The test pilot used a 3-DoF joystick for teleoperation (requiring switching between three modes) and a touchscreen to select objects of interest. All test pilots had a one-hour training session to practice operating the robot via teleoperation as well as via the VGS control. After the training, the test pilot was asked to perform the drinking task, first with teleoperation for five trials, and then with VGS control for five trials. The robotic arm started from the home position for each trial. Any part of the task that was not completed was recorded as a failure and the user was instructed to continue with the remaining parts. For example, if the cabinet failed to open, it was opened so the user could access the cup for the next subtask. For each trial, we recorded the task completion status (success or failure), time of completion in seconds, and joystick mode-switch frequency for each subtask. We used the criteria in Table 1 for both teleoperation and VGS trials.

We also performed an evaluation with a case participant who has a C6 incomplete spinal cord injury. The participant met the inclusion criteria: using a power wheelchair as a primary means of mobility and self-reporting to have difficulties in performing everyday manipulation tasks such as reaching for a glass of water, opening a refrigerator, and picking up a toothbrush. The Institutional Review Board of the VA Pittsburgh Healthcare System approved the protocol. Informed consent was obtained prior to the start of the study. The experiment setup and protocol were similar to the test pilot evaluation, except that the participant sat to the left of the ARM, used a 2-DoF joystick and two external switches (requiring switching between four modes) to operate the robotic arm, and was asked to perform two trials of teleoperation and VGS control, respectively. The participant was also asked to rate the perceived workload he experienced for each control method via the NASA Task Load Index (NASA-TLX) [26] and the perceived ease-of-use via the System Usability Scale (SUS) [27]. The NASA-TLX has been shown to be valid and has excellent test–retest reliability [28]. It consists of six dimensions (mental demands, physical demands, temporal demands, performance, efforts, and frustration), and each dimension was rated by the participant using twenty-step bipolar scales; this resulted in a score between 0-100, with a higher score indicating a higher workload for that dimension. The SUS provides a global measure of user satisfaction and has also been shown to be reliable and valid. It consists of 10 statements that the participant was asked to rate on a 5-point Likert scale (0—strongly disagree to 4—strongly agree), resulting in a total score between 0–100, with a higher score indicating better usability and overall satisfaction [27].

## 3. Results

The bench-top test results are shown in Table 2, including the success rates and failure descriptions for each autonomous action of the drinking task under the VGS control. Opening the cabinet from an arbitrary initial gripper orientation had the lowest success rate. One common reason for all failed operations was a loose grip, where the gripper was unable to hold the door handle or cup firmly and lost its grip during the movement. This was possibly due to errors in localizing the fiducial tags, especially when the gripper was in arbitrary initial orientations. Another major failure was in path planning, including unnatural paths from the sample-based planning approach, collision, and failure to find a feasible path.

The results from the three test pilots (TP #1 & TP #2: limited experience; TP #3: significant experience) over five trials with each control method are shown in Table 3, Table 4, Table 5 and Table 6 for the subtask success rate, time spent, and 3-DoF joystick mode-switch frequency, respectively. Table 6 shows the overall task-performance comparison between the teleoperation and VGS control.

**Figure 6:**
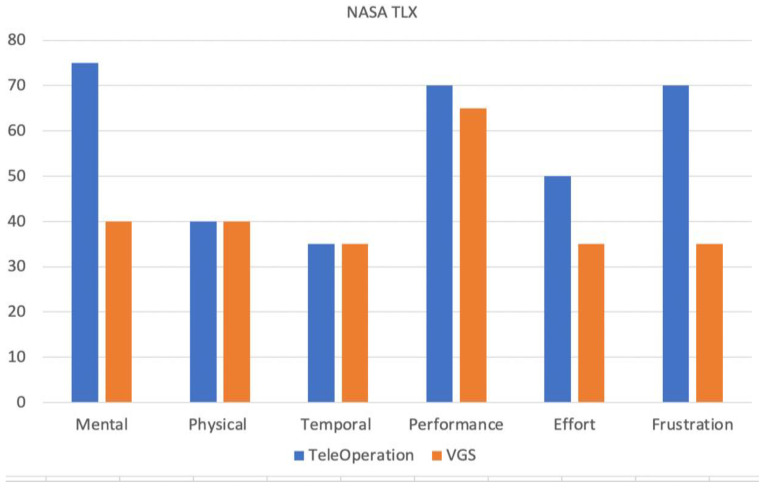
NASA-TLX ratings for both control methods by the case participant.

The case participant was a 36-year-old male with C-6 incomplete spinal cord injury. He chose to use a 2-DoF joystick with two external switch buttons placed on the armrest of his power wheelchair. His performance with teleoperation and VGS is shown in Table 7 and Table 8. In addition, his ratings on the six dimensions of the NASA-TLX for both control methods are shown in Figure 6. He rated the usability of teleoperation with a SUS of 70 and the usability of VGS with a SUS of 72.5.

## 4. Discussion

We described the development and preliminary implementation of a new shared-control approach for ARMs in this paper. This work was built upon our previous work [19,29] and expanded the implementation of VGS control from addressing simple one-step tasks to addressing multi-step functional tasks. We observed that the VGS control delivered similar benefits for multi-step functional tasks as for simple one-step tasks, in terms of improving overall task performance for test pilots and the case participant and reducing perceived workload for the case participant.

In terms of success rates, from Table 3 and Table 7, opening the cabinet was the most challenging subtask for all individuals regardless of their experience with operating a robotic arm. This is similar to the findings by Kadylak et al. [30], who found that objects that rotate about a fixed axis were the most difficult to manipulate when teleoperating a mobile manipulator. As a joystick cannot control more than two DoFs at a time, it is difficult to control the end-effector to follow a curved trajectory. Even with the autonomous action of the VGS control, we had to compose five waypoints along a 75-degree arc trajectory from the door hinge and handle location based on the position and orientation of the cabinet fiducial tag, so the robot was able to pull the cabinet open smoothly. The autonomous action for this subtask helped improve the success rates, as shown in Table 3 and Table 7; however, it still fell short on two occasions, primarily due to pose estimation errors of the fiducial tag. Table 1 also indicates a similar issue with the fiducial tag.

In terms of time spent, from Table 6 and Table 8, the overall time spent on the drinking task was reduced by 14–51% across all individuals under the VGS control. It was also interesting to see that VGS helped equalize performance regardless of the initial skills among the three test pilots, indicating that VGS could help novice users to become acquainted with the robot quickly and potentially increase technology acceptance, especially for those who are less technologically inclined. We also observed that the time reduction for the overall task with VGS was mostly due to the subtask of opening a cabinet, where the time reduction ranged from 41–72% across the participants (Table 4 and Table 7). For other subtasks, VGS sometimes took longer than teleoperation. For example, the subtask of retrieving a cup was slower with VGS for two test pilots. Given that this subtask is relatively simple, it is possible that some users could be faster by directly approaching and grasping the cup via teleoperation than with VGS, which involves locating the fiducial tag first and waiting for the autonomous action to find and execute a path to grasp the cup. The subtask of filling the cup also saw no time improvement with almost all participants. This subtask has an autonomous action (Table 1) that brings the robot close to and aligns it with the dispenser tap, and a teleoperation action that requires the user to move the robot to push the jug dispenser tap. More time spent with VGS could be attributed to participants not starting the teleoperation right after the autonomous action, especially if they did not pay full attention to the task progress. We observed that participants were less focused during the VGS control and sometimes initiated conversations while waiting for the robot to carry out its work. One participant commented that she could converse with others while using the VGS control to complete a task but would not be able to do so when using teleoperation. It is interesting to note that time of completion may not be the best metric for evaluating robot performance in this context. Finally, we also observed that the case participant sometimes forgot to use the VGS control, especially for the last two subtasks, where the autonomous actions require the user to press a button on the touchscreen instead of moving the robot to find a fiducial tag. The case participant forgot to activate the autonomous actions on several occasions and ended up completing the task via teleoperation during the VGS control.

It is worth noting that the success rates and time of completion should be considered together for performance assessment, and any one measure may not be sufficient to reflect the true performance. For example, the third test pilot (TP #3) had significantly more experience with operating a robotic arm than the other two test pilots. As shown in Table 4 (teleoperation time which more accurately reflects the ability and skill of a person to operate the robot), the time achieved by TP #3 was, in general, the shortest; the exception was for the step ‘fill cup’, where he spent more time carefully positioning the cup to avoid spills, while TP #1 did not consider this. However, in terms of success rates, TP #3 had a lower rate for the ‘open cabinet’ step. Given that this was the most challenging step, his increased task speed may have caused an unintended failure. We also observed that some participants developed competitiveness and cared less about being successful and more about speed.

In terms of joystick mode-switch frequency, there is a very clear trend in which the VGS control required less-frequent switches between different joystick modes than teleoperation, irrespective of the type of subtask. With the 3-DoF joystick, the test pilots did not even need to switch joystick modes on many occasions. When a user does not need to switch between joystick modes frequently, there should be minimal mental workload to perform a task. This is consistent with the comments and observations we mentioned above, which state that participants did not pay full attention during the VGS control, and also with the NASA-TLX ratings from the case participant in Figure 6. The perceived workload in four out of six dimensions of the NASA-TLX were reduced with the VGS control, including the mental demand (how mentally demanding was the task), performance (how successful the participant was in accomplishing the task), effort (how hard one must work), and frustration (how irritated and stressed the participant was). The physical demand and temporal demand did not differ between the two methods, as the case participant still had to physically teleoperate the robot during the VGS control and did not feel rushed when testing both control methods. Finally, when examining the time spent and joystick mode-switch frequency, we also observed that the standard deviation of these variables across multiple trials under VGS control was much smaller than under teleoperation, indicating that user performance became more reliable and consistent with VGS.

While the VGS control showed initial promise based on task performance and perceived workload, there were some challenges during implementation. One challenge is to address the control-authority transition between the robot and the user so they can collaborate effectively to complete the tasks. We chose to use a touchscreen which displays a GUI for the user to select targets of interest and view task-progress messages. While this method worked in general, users sometimes did not pay attention and missed the GUI messages, and required verbal cues to continue. The control-authority transition in the current VGS implementation was also pre-defined, and thus, there was no flexibility for users to override the system. Another challenge for implementing the VGS control for multi-step functional tasks is in manipulator path planning. Multi-step tasks typically involve multiple objects in the scene, and all objects are considered obstacles during manipulator path planning. Finding a collision-free path with constraints (e.g., keeping the cup level) in a cluttered environment in a short period of time is still an open research topic in the robotic field [31]. We observed that it sometimes took several tries to find a valid path in our allocated five-second planning time. In some cases, no path was found, or the path moved in unintuitive ways due to the sample-based planner selected. The path planning failures may also be attributed to the accuracy of obstacle modeling in the environment based on the fiducial tags. While the ArUco tags we used have great detection rates, errors in position and orientation measurements may have added uncertainty that increased failure rates for autonomous actions that require more precision. These challenges led to some usability issues during the VGS control. The case participant rated the usability of VGS slightly higher than teleoperation (SUS: 72.5 vs. 70). While he recognized that it was easier for him to learn and actually use the VGS control, he also found that the VGS control was a bit unnecessarily complex and inconsistent as compared to teleoperation.

This study has several limitations. First, the ‘fill cup step was performed in a simulated way to avoid accidental spills due to overflow or inappropriate cup positioning, which makes it difficult to accurately determine task success. Second, we only evaluated the VGS control against teleoperation in three test pilots without disability and one participant with disability. While the results cannot be generalized, they provide more insights on the feasibility of the VGS control and help inform future studies that will enroll more participants with disability in the evaluation of VGS effectiveness. Third, we mounted the robot on a table instead of a power wheelchair as the robot would be used in real-life conditions. Mounting the robot on a power wheelchair could introduce significant challenges for localizing target objects due to the lack of a fixed reference. In addition to the power wheelchair movements in the space needing to be tracked, the attachment point of the robot arm’s base position should also be tracked, as the user’s movements in the wheelchair and wheelchair seating functions could affect it. Fourth, we only demonstrated the VGS control with one multi-step functional task in this study, and each new task would need to be programmed. However, our software architecture was designed to allow re-use of the same manipulation types for different tasks. For example, a pick sequence is used for grasping the cup in the cabinet in the drinking task. This same sequence could retrieve any object and contains the following actions: open gripper, reach to object, grasp object, and retract with object. Similarly, a push sequence closes the gripper, reaches to a goal position, moves in a push motion, and then retracts in the opposite direction. This sequence was used when filling the cup, and could generalize to subtasks such as opening a microwave. As new manipulation types such as pour and twist are covered in the software system, programming a new task will become easier. Fifth, the VGS control lacked robustness. In addition to the aforementioned fiducial tag localization errors that led to loose grips on several occasions, another rare failure occurred for the case participant when he placed the cup back on the table under the VGS control. The GUI crashed due to a threading issue, causing the participant to lose track of the joystick mode displayed on the GUI. He accidentally opened the gripper and dropped the cup when trying to move the cup away from his mouth and back to the table. Lastly, the test pilots performed five trials for each step of the task, and the case participant performed only two trials. The low number of trials makes it difficult to draw any significant conclusions. For the case participant, while the VGS reduced his task completion time and mode-switch frequency, he failed once during teleoperation and during VGS, respectively, resulting in only a 50% success rates for both control methods. Increasing the number of trials might be helpful to assess the system performance more accurately and provide an opportunity to observe the learning/training effect.

Future work will focus on improving different components of the VGS control, including perception, path planning, and control-authority transition between the user and the robot. The results from this study indicate that some manipulation types, such as pulling along a curved trajectory, benefit more from the autonomous actions of the VGS control than others. As more functional tasks are considered, it is helpful to investigate how potential users of ARMs would like to collaborate with the robot to accomplish tasks with varying manipulation types. Future work should also extend the VGS control to more realistic settings such as mounting the robot to a power wheelchair or a mobile platform. The robot could also become more intelligent by adding sensors on the gripper and in the environment to achieve context awareness, so it could more properly respond to task failures such as retrying a task on its own or enlisting help from the user when needed.

## 5. Conclusions

This study presented the preliminary implementation of a new shared-control approach—VGS control—that combines autonomous actions and user teleoperation for supporting complex multi-step tasks that are not commonly addressed in assistive robotics literature. The VGS control showed the potential to improve task-performance parameters such as success rate, joystick mode-switch frequency, and time of completion for certain manipulation types; improve performance consistency; and reduce cognitive workloads. Improvements are needed to test the system with more potential users on a variety of complex multi-step functional tasks; nevertheless, VGS control could potentially serve as a platform for investigating different human–robot interaction strategies, as well as guiding meaningful technology-development for each component of the system in the context of supporting meaningful functional tasks for people with disabilities.

## Figures and Tables

**Table 1:** How a user and a robot work together under VGS control for a multi-step drinking task.

	Success Criteria	Teleoperation Actions by User	Autonomous Actions by Robot
Open cabinet	The cabinet door is fully open and stays open.	Move the robot to find the tag on the cabinet.	Grasp the cabinet handle and pull the cabinet open fully.
Retrieve cup	The cup stays upright and firmly held in the gripper, and is lifted above the surface.	Move the robot to find the tag on the cup.	Grasp the cup and lift it up.
Fill cup	The jug dispensing-tap is pushed back by the cup, which stays about upright. (This was performed in a simulated way in which no water was dispensed, to avoid accidental spill or overflow. Thus, the amount of water and water spill were not considered in the criteria).	Move the robot (with cup in hand) to find the tag on the jug Push the cup against the dispenser tap on the jug to fill and then move away from jug.	Move close to and align the cup with the dispenser tap on the jug.
Drink	The cup stops at a position feasible for drinking from it and remains upright during transport.	Move the robot to a drinking position based on individual needs and drink from cup.	Move the robot to a default drinking position.
Place cup on table	The cup is placed on the table and stays upright.	Move the robot back towards the table.	Place the cup back on the table.

**Table 2:** Bench-top test results of autonomous actions under VGS control.

Autonomous Action	Success	Failure Descriptions (# of Failed Trials)
Open cabinet (fixed initial gripper-orientation)	85%	Loose grip (1) Unnatural path led to collision when approaching handle (1) Failed to find a path to reach the cabinet handle (1)
Open cabinet (arbitrary initial gripper-orientation)	80%	Loose grip (3) Unnatural path led to collision when approaching handle (1)
Retrieve cup	85%	Loose grip (1) ARM scratched table (1) ARM collided with cabinet door (1)
Fill cup	90%	Cup collided with the table (2)
Drink	95%	Unnatural path led to inappropriate cup orientation (1)
Place cup on table	100%	None

**Table 3:** Subtask success rate during teleoperation and VGS control.

	Open Cabinet		Retrieve Cup		Fill Cup		Drink		Place Cup	
	Tele	VGS	Tele	VGS	Tele	VGS	Tele	VGS	Tele	VGS
TP #1	80%	100%	100%	100%	100%	100%	100%	100%	100%	100%
TP #2	60%	80%	80%	100%	100%	100%	100%	100%	100%	100%
TP #3	60%	80%	100%	80%	100%	100%	100%	100%	100%	100%

**Table 4:** Subtask time spent in seconds during teleoperation and VGS control (standard deviation was over five trials).

	Open Cabinet		Retrieve Cup		Fill Cup		Drink		Place Cup	
	Tele	VGS	Tele	VGS	Tele	VGS	Tele	VGS	Tele	VGS
TP #1	84.7 ± 16.6	27.8 ± 0.5	22.3 ± 10.6	27.4 ± 3.6	13.9 ± 7.6	52.3 ± 8.9	36.2 ± 10.1	17.2 ± 2.7	31.9 ± 13.9	12.5 ± 0.0
TP #2	116.7 ± 41.4	33.1 ± 9.2	43.1 ± 7.3	23.7 ± 3.0	40.3 ± 28.4	39.1 ± 3.3	44.1 ± 5.5	27.7 ± 4.8	27.8 ± 6.1	10.6 ± 0.7
TP #3	57.9 ± 43.4	33.9 ± 13.4	19.6 ± 3.7	25.5 ± 5.8	24.7 ± 4.5	39.3 ± 8.2	29.4 ± 2.6	16.2 ± 3.8	16.2 ± 1.7	12.6 ± 0.1

**Table 5:** Subtask joystick mode-switch frequency during teleoperation and VGS control.

	Open Cabinet		Retrieve Cup		Fill Cup		Drink		Place Cup	
	Tele	VGS	Tele	VGS	Tele	VGS	Tele	VGS	Tele	VGS
TP #1	8.2 ± 2.3	0 ± 0	4.4 ± 3.6	0 ± 0	0 ± 0	0 ± 0	2 ± 0	1.2 ± 0.5	2.6 ± 1.5	1.2 ± 0.5
TP #2	16.2 ± 5.3	0 ± 0	7.2 ± 1.3	0 ± 0	2.8 ± 2.3	0 ± 0	7 ± 1.4	1.2 ± 0.5	4.4 ± 1.5	1.2 ± 0.5
TP #3	6.0 ± 3.0	0 ± 0	3.6 ± 0.9	0 ± 0	2.2 ± 0.5	0 ± 0	2 ± 0	1.2 ± 0.5	1.0 ± 0.0	1.4 ± 0.6

**Table 6:** Overall task performance during teleoperation and VGS control.

	Success Rate		Time Spent (s)		Mode Switch	
	Tele	VGS	Tele	VGS	Tele	VGS
TP #1	80%	100%	189.0 ± 16.7	137.2 ± 8.8	17.2 ± 4.8	2.4 ± 0.9
TP #2	60%	80%	271.9 ± 38.2	134.2 ± 13.6	37.6 ± 7.9	2.4 ± 0.9
TP #3	60%	80%	147.8 ± 43.7	127.5 ± 18.1	14.8 ± 3.7	2.6 ± 0.9

**Table 7:** Subtask success rate, time spent in seconds, and joystick mode-switch frequency for the case participant.

	Open Cabinet		Retrieve Cup		Fill Cup		Drink		Place Cup	
	Tele	VGS	Tele	VGS	Tele	VGS	Tele	VGS	Tele	VGS
Success	50%	100%	100%	100%	100%	100%	100%	100%	100%	50%
Time (s)	132.6 ± 64.0	45.4 ± 9.3	59.9 ± 13.4	53.1 ± 13.1	39.1 ± 7.7	55.2 ± 12.2	50.9 ± 0.0	70.1 ± 0.1	47.8 ± 1.9	58.3 ± 7.7
Mode Switch	24.5 ± 10.6	1.5 ± 0.7	10.5 ± 0.7	3.0 ± 2.8	7.0 ± 1.4	4.5 ± 2.1	7.5 ± 5.0	7 ± 1.4	7.5 ± 2.1	6.5 ± 0.7

**Table 8:** Overall task performance for case participant for two trials.

	Success Rate		Time Spent (s)		Mode Switch	
	Tele	VGS	Tele	VGS	Tele	VGS
Case	50%	50%	330.3 ± 83.3	282.1 ± 24.7	57 ± 11.3	22.5 ± 2.1

## Data Availability

The data presented in this study are available on request from the corresponding author. The data are not publicly available due to being restored by the US Department of Veterans Affairs, and are subject to the approval of the relevant authority.

## References

[1] Chen T.L., Ciocarlie M., Cousins S., Grice P.M., Hawkins K., Hsiao K., Kemp C.C., King C.-H., Lazewatsky D.A., Leeper A.E. (2013). Robots for humanity: Using assistive robotics to empower people with disabilities. IEEE Robot. Autom. Mag..

[2] Brose S.W., Weber D.J., Salatin B.A., Grindle G.G., Wang H., Vazquez J.J., Cooper R.A. (2010). The role of assistive robotics in the lives of persons with disability. Am. J. Phys. Med. Rehabil..

[3] Herlant L.V., Holladay R.M., Srinivasa S.S. Assistive Teleoperation of Robot Arms via Automatic Time-Optimal Mode Switching. Proceedings of the 2016 11th ACM/IEEE International Conference on Human-Robot Interaction (HRI).

[4] Kemp C., Edsinger A., Torres-Jara E. (2007). Challenges for robot manipulation in human environments [Grand Challenges of Robotics]. IEEE Robot. Autom. Mag..

[5] Chung C.S., Wang H., Cooper R.A. (2013). Functional assessment and performance evaluation for assistive robotic manipulators: Literature review. J. Spinal Cord Med..

[6] Chung C.S., Ka H.W., Wang H., Ding D., Kelleher A., Cooper R.A. (2017). Performance Evaluation of a Mobile Touchscreen Interface for Assistive Robotic Manipulators: A Pilot Study. Top Spinal Cord Inj. Rehabil..

[7] Losey D.P., Srinivasan K., Mandlekar A., Garg A., Sadigh D. Controlling Assistive Robots with Learned Latent Actions. Proceedings of the 2020 IEEE International Conference on Robotics and Automation (ICRA).

[8] Beaudoin M., Lettre J., Routhier F., Archambault P.S., Lemay M., Gélinas I. (2019). Long-term use of the JACO robotic arm: A case series. Disabil. Rehabil. Assist. Technol..

[9] Pulikottil T.B., Caimmi M., Dangelo M.G., Biffi E., Pellegrinelli S., Tosatti L.M. A Voice Control System for Assistive Robotic Arms: Preliminary Usability Tests on Patients. Proceedings of the IEEE RAS and EMBS International Conference on Biomedical Robotics and Biomechatronics.

[10] Aronson R.M., Admoni H. Semantic gaze labeling for human-robot shared manipulation. Proceedings of the 11th ACM Symposium on Eye Tracking Research & Applications.

[11] Admoni H., Srinivasa S. (2016). Predicting User Intent through Eye Gaze for Shared Autonomy. Proceedings of the 2016 AAAI Fall Symposium Series: Shared Autonomy in Research and Practice.

[12] Andreasen Struijk L.N.S., Egsgaard L.L., Lontis R., Gaihede M., Bentsen B. (2017). Wireless intraoral tongue control of an assistive robotic arm for individuals with tetraplegia. J. Neuroeng. Rehabil..

[13] Collinger J.L., Wodlinger B., Downey J.E., Wang W., Tyler-Kabara E.C., Weber D.J., McMorland A.J.C., Velliste M., Boninger M.L., Schwartz A.B. (2013). High-performance neuroprosthetic control by an individual with tetraplegia. Lancet.

[14] Ranganathan R., Lee M.H., Padmanabhan M.R., Aspelund S., Kagerer F.A., Mukherjee R. (2019). Age-dependent differences in learning to control a robot arm using a body-machine interface. Sci. Rep..

[15] Ivorra E., Ortega M., Catalán J.M., Ezquerro S., Lledó L.D., Garcia-Aracil N., Alcañiz M. (2018). Intelligent multimodal framework for human assistive robotics based on computer vision algorithms. Sensors.

[16] Jain S., Argall B. (2020). Probabilistic Human Intent Recognition for Shared Autonomy in Assistive Robotics. ACM Trans. Hum.-Robot Interact..

[17] Driessen B.J.F., Liefhebber F., Kate T.T.K.T., Van Woerden K., Ten Kate T., Van Woerden K. Collaborative Control of the MANUS Manipulator. Proceedings of the 9th International Conference on Rehabilitation Robotics, 2005 (ICORR 2005).

[18] Gopinath D., Jain S., Argall B.D. (2017). Human-in-the-Loop Optimization of Shared Autonomy in Assistive Robotics. IEEE Robot. Autom. Lett..

[19] Ka H.W., Chung C.S., Ding D., James K., Cooper R. (2018). Performance evaluation of 3D vision-based semi-autonomous control method for assistive robotic manipulator. Disabil. Rehabil. Assist. Technol..

[20] Kalaitzakis M., Cain B., Carroll S., Ambrosi A., Whitehead C., Vitzilaios N. (2021). Fiducial Markers for Pose Estimation. J. Intell. Robot. Syst..

[21] Romero-Ramirez F.J., Muñoz-Salinas R., Medina-Carnicer R. (2018). Speeded up detection of squared fiducial markers. Image Vis. Comput..

[22] Garrido-Jurado S., Muñoz-Salinas R., Madrid-Cuevas F.J., Medina-Carnicer R. (2016). Generation of fiducial marker dictionaries using Mixed Integer Linear Programming. Pattern Recognit..

[23] Gorner M., Haschke R., Ritter H., Zhang J. MoveIt! Task Constructor for Task-Level Motion Planning. Proceedings of the 2019 International Conference on Robotics and Automation (ICRA).

[24] Coleman D., Sucan I., Chitta S., Correll N. (2014). Reducing the Barrier to Entry of Complex Robotic Software: A MoveIt! Case Study. J. Softw. Eng. Robot..

[25] Kuffner J.J., LaValle S.M. RRT-connect: An efficient approach to single-query path planning. Proceedings of the 2000 ICRA Millennium Conference, IEEE International Conference on Robotics and Automation, Symposia Proceedings (Cat No00CH37065).

[26] Hart S.G., Staveland L.E. (1988). Development of NASA-TLX (Task Load Index): Results of Empirical and Theoretical Research. Adv. Psychol..

[27] Brooke J. (1996). SUS—A quick and dirty usability scale. Usability Evaluation in Industry.

[28] Devos H., Gustafson K., Ahmadnezhad P., Liao K., Mahnken J.D., Brooks W.M., Burns J.M. (2020). Psychometric properties of NASA-TLX and index of cognitive activity as measures of cognitive workload in older adults. Brain Sci..

[29] Ka H.W., Ding D., Cooper R. (2016). Three Dimensional Computer Vision-Based Alternative Control Method For Assistive Robotic Manipulator. Int. J. Adv. Robot. Autom..

[30] Kadylak T., Bayles M.A., Galoso L., Chan M., Mahajan H., Kemp C.C., Edsinger A., Rogers W.A. (2021). A human factors analysis of the Stretch mobile manipulator robot. Proc. Hum. Factors Ergon. Soc. Annu. Meet..

[31] Cohen B.J., Subramania G., Chitta S., Likhachev M. Planning for Manipulation with Adaptive Motion Primitives. Proceedings of the 2011 IEEE International Conference on Robotics and Automation.

